# When all doors close: Implications of COVID-19 for cosmopolitan entrepreneurs

**DOI:** 10.1177/0266242620954127

**Published:** 2020-12

**Authors:** Niina Nummela, Eriikka Paavilainen-Mäntymäki, Riikka Harikkala-Laihinen, Johanna Raitis

**Affiliations:** University of Turku, Finland

**Keywords:** cosmopolitan, cosmopolitan entrepreneur, cosmoscape, COVID-19, mobility

## Abstract

The emergence of the COVID-19 pandemic is an external shock that has disrupted the foundations of everyday life. For cosmopolitan entrepreneurs, the impact is even more decisive as it confronts their core values and jeopardises their identities, ways of working and the lifestyles they cherish. Cosmopolitans are individuals who identify themselves as citizens of the world and voluntarily move from country to country in pursuit of self-fulfilment in both life and work. Cosmopolitan entrepreneurs are future-oriented and open to the world and the opportunities it may provide. Beyond securing, maintaining and improving their professional and/or economic positions, their mobility is an elementary part of the cosmopolitan life itself, something they find attractive, interesting and stimulating. Thus, a cosmopolitan entrepreneur’s business is often non-location-bound to enable continued mobility. With our interview-based research, we shed light on how COVID-19 has changed the lives of Finnish-born cosmopolitan entrepreneurs, discussing what they feel about the changes and how they see their future.

## Emergence of the Black Swan: COVID-19

Although there have been a number of pandemics throughout human history, only the latest, COVID-19, can be labelled a ‘Black Swan’ (see Taleb, 2007). The virus itself is neither rare nor unpredictable, but its impact certainly is – COVID-19 has imposed social distancing, lockdowns and restrictions on mobility with immediate effects on societies around the globe, such as a dramatic reliance on the conveniences of online shopping, social media use and teleconferencing (World Trade Organization, 2020). International travel bans at one point affected more than 90% of the world population with widespread restrictions on public events and an outright cessation of tourism in many cases (Gössling et al., 2020). Almost overnight, the globally connected world transformed into a stay-at-home economy (World Economic Forum, 2020); it is likely that international travel – particularly nonessential movements – will remain challenging for some time to come, and that these restrictions will persist for the foreseeable future (Benton, 2020). Social distancing, international travel bans and stay-at-home recommendations are particularly challenging for cosmopolitan individuals who value freedom, openness and mobility. By definition, geography, place, countries and other traditional location-based characteristics do not limit how they live, work, venture, experience and learn when moving from country to country in pursuit of self-fulfilment in both life and work, and constructing a cosmopolitan identity in the process (Nummela et al., 2021; Raitis et al., 2019).

Cosmopolitan orientation has been increasing despite the global business environment being volatile, uncertain, complex and ambiguous (Schoemaker et al., 2018; Van Tulder et al., 2019) and although anti-globalisation tendencies have gained momentum alongside global interconnectedness and interdependencies (Meyer, 2017). In light of these trends, the impact of COVID-19 has resulted in an economic crisis far different from previous, financial-based ones. Among its key features is its focus on individuals (Cortez and Johnston, 2020). Its consequences are frequently seen at the grassroots level as individuals experience forms of crisis and vulnerability differently (Perrig-Chiello et al., 2016). Thus, the emerging ‘new normal’ is a subjective experience and a challenge for all. Recovering from the external shock of a global pandemic will be particularly demanding for cosmopolitan entrepreneurs. International lockdowns and the rise of a stay-at-home economy confront their core values and jeopardise the identities and lifestyles they cherish. In this commentary, we study how COVID-19 has changed the life of Finnish-born cosmopolitan entrepreneurs, what they feel about those changes and how they see their future.

## Cornerstones of being a cosmopolitan entrepreneur

Cosmopolitan individuals view themselves as world citizens (Bayram, 2015) moving from country to country in pursuit of self-fulfilment in both life and work, constructing a cosmopolitan identity in the process (Nummela et al., 2021). Geography, place, countries and other traditional, location-based characteristics do not limit how cosmopolitans live, work, venture, experience and learn (Raitis et al., 2019). They are attracted to *cosmoscapes* – spaces, practices, objects and networks facilitating cosmopolitan life (Kendall et al., 2009) – which downplay national affiliations and cultural differences (Skovgaard-Smith and Poulfelt, 2018). The key characteristics that define a cosmopolitan individual are mobility, openness, a valuing of different cultures and a disengagement from national and local anchors (Skrbiš and Woodward, 2013). For cosmopolitans, mobility itself is empowering, and it provides them with the means to live a life they prefer (Nummela et al., 2021); however, it is not required to maintain their profession or finances (cf. Loacker and Śliwa, 2016). This mode of belongingness – or rather, a conspicuous lack of belonging – distinguishes cosmopolitans from other migrating and mobile groups (Skovgaard-Smith and Poulfelt, 2018). They often find themselves in-between; this liminal experience is due to ‘being located yet mobile’ (Daskalaki, 2012; Daskalaki et al., 2016).

Cosmopolitans differ from other individuals in their sense of place^1^ and time (Brimm, 2010), and this is reflected in their life patterns, identities and behaviours as they relate to work, entrepreneurship and communication. As entrepreneurs, cosmopolitans search, identify, create and exploit business opportunities widely, and these processes are not bound to a specific geographical area (Marotta, 2010). Their interests instead focus on local opportunities outside their conventional experience (Vertovec and Cohen, 2003). Because of this, the entrepreneurial path of a cosmopolitan entrepreneur may look quite sporadic and their businesses multifocal and multilocal (see Elo et al., 2019). Interestingly, cosmopolitans often have characteristics that are typically associated with entrepreneurs, including a high need for achievement (McClelland, 1965) and tolerance of risk (Lumpkin and Dess, 1996), but also hold capabilities that facilitate both the discovery and creation of entrepreneurial opportunities (Nummela et al., 2021). In addition, they are adept at boundary spanning, in particular if they are both professionally competent and possess good networking capabilities (cf. Tushman and Scanlan, 1981). Many cosmopolitan entrepreneurs benefit from the use of technology, allowing them to work wherever they want, but not all cosmopolitan entrepreneurs are ‘digipreneurs’.

During this pandemic, we have all experienced shared fear, often leading to decreased psychological safety (Edmondson, 2020). We assume that these decreases have been particularly notable among cosmopolitan entrepreneurs whose lives have been built on openness and mobility in both the private and occupational spheres. For example, some cosmopolitans earn their living as travel bloggers. Now that travel for leisure is greatly reduced, there is little need for such services, and it is questionable whether our use of their content will ever rise to previous levels.

## How has COVID-19 changed the lives of cosmopolitan entrepreneurs?

In our ongoing study on cosmopolitans,^2^ we have been able to follow their lives during the COVID-19 crisis. Naturally, the most important feature of the pandemic for them has been the loss of mobility. This creates mixed feelings among cosmopolitans: on one hand, stress levels remain lower because of reduced travel; on the other, many of them describe the lockdown as ‘*being in jail’*; one even used the expression ‘*end of the world*’. Many – but not all – have returned to their countries of origin – for safety reasons – something for which they had not planned.

Many cosmopolitan entrepreneurs worked online before the pandemic, so in terms of professional outlook, the lockdown has not been a major change. However, developing businesses online, and particularly exploring and exploiting new opportunities, is difficult or at least requires learning new skills. Overall, although the businesses of our cosmopolitans were affected, they will survive. One of them stated,

‘Year 2020 was supposed to be a year of growth now it has turned into a year of perseverance’. (KH)

The major impacts of the pandemic can best be observed at the individual level. In these times, family relations and station in life make a significant difference; for single cosmopolitans, daily life is quite lonely. They keep in touch with their transnational families, friends and business connections around the world, a process aided by the fact that they are practiced users of online tools. Cosmopolitans with families experience the lockdown in a similar way as others – extensive stay-at-home periods with their children. It seems that the emergence of the pandemic has strengthened the connections in their personal and business networks:

The pandemic has also changed my social interactions with family and friends – I rely on remote communications. ‘Keeping in touch’ and ‘checking in’ has intensified significantly, especially with XXX colleagues who are in field locations, either stuck behind closed borders or who are otherwise in difficult circumstances. Physical remoteness has brought us closer together. (KV)

Feelings about the pandemic and related restrictions are mixed. Positive emotions are mixed with negative ones, and they change often; lower levels of travel have a positive impact in terms of both personal schedules and protecting the environment. The compulsory travel ban has also generated greater tranquillity and relief among some cosmopolitans:

I have felt positive all the time, and in a way, the quality of life has improved. I have had time to stop, think about the future, and make long-term plans instead of constantly living out of a suitcase, in the present. Before the pandemic, I was searching for my place in the world; now, I have found it. (OL)

One crisis triggers other crises. Many think about their life choices and want to change them. Compulsory halting, that feeling of helplessness in front of the virus and the reminder of one’s mortality, are radical experiences. . . . I have thought about that tale in the Bible, how the one built his house on sand and the one on the rock. When the storm came, the house on the rock stood, whereas the other was in ruins. I have gone through similar feelings – grief and disappointment – as probably the majority of other people have. At the same time, I have been happy that my own life is not based on material things or status. I do not have to be afraid of decreasing living standards or not being acknowledged. And yet, I have had to practice giving up and it hurts. (KH)

Conversely, a growing focus upon psychological safety among the interviewees is evident. For cosmopolitans whose networks reach across the globe, anxiety is not limited to themselves but also to family, friends and colleagues in different parts of the world. This is reflected in their emotional status, as happiness can often be mixed with guilt with the knowledge that others are not as safe as they are:

This is yet another reminder of life not being about rigid plans, no matter how little we like uncertainty. As I am due to return to work sometime in July, I am faced with lots of uncertainty with timing, as there is little clarity regarding relocation options with travel restrictions. I have to be ready for potential changes even in the next assignment. Keeping an open mind is the key. Considering the fate and state of some of my colleagues in the deep field right now, I am probably in the best possible location I could be – at home, close to my friends and family, close to good healthcare, and safe from any additional hazards present in the third world countries, including being a target of ‘extra hatred’ for being a foreigner. All in all, I am damn lucky. (KV)

What next? Some of our cosmopolitans are eagerly waiting for a return to the ‘new normal’, for borders to open and restrictions to lift so that they can travel back to their homes outside their countries of origin. Some explicitly state that they do not want to change their lives but suspect that keeping their previous lifestyles will require novel solutions. A few are at a watershed: the pandemic is a critical event which has led them to rethink their values and lives. A permanent change of lifestyle lies ahead for some:

COVID-19 highlighted a lack of permanence in my life and how important it is to be prepared for a rainy day, in particular financially. Since the pandemic started, I have started planning for strategic saving. . . . Overall, I have settled down, stopped to think, and learned where my home is. (OL)

The standstill has also been a trigger for some entrepreneurs to think about the capabilities and skills needed in the future and for starting new projects, such as writing a book or realising long-term dreams. One of our interviewees stated, ‘The inward turn has triggered a feeling that “the future is now”’ (MHS).

## Discussion

Although the findings from our interviews are tentative, they are interesting and suggest that we might be on the verge of a major change. Is our open and globally connected world turning into a more closed one? We have seen indications of it previously in the anti-globalisation debate (Meyer, 2017), but the pandemic has brought the issue to the level of individual citizens. It is possible that the changes we have recently experienced will have permanent effects on how cosmopolitan entrepreneurship is practised in the future. Cosmopolitans are often described as mobile and open (Skrbiš and Woodward, 2013). It is natural that, due to the pandemic, cosmopolitans are now less mobile. However, the lockdown seems to have given rise to other, more fundamental consequences. Our interviews indicate that, after the emergence of the pandemic, these cosmopolitans may not be as open to opportunities as before. However, this finding is only tentative, as the interviews were conducted in early spring 2020, and it is possible that these first reactions were stronger and the views more pessimistic than they later will be when the situation normalises. In the future, entrepreneurial opportunities will be evaluated from the viewpoint of what new skills are needed and where it will be safest to exploit them. The current situation is stressful for a cosmopolitan entrepreneur who needs to sustain a competitive business and at the same time protect their health (Ratten, 2020). Cosmopolitan entrepreneurs – as with all entrepreneurs – have entered into ‘survival mode’ with resilience and ambidexterity and are doing their best to secure business continuity. However, it may be that the types of business opportunities they have previously exploited will no longer exist in the future.

On the positive side, cosmopolitans are known to be flexible and tolerant of uncertainty. Furthermore, they are competent in using virtual tools to support their businesses and personal lives. Thus, they may have an advantage as more work moves to virtual spaces and flexible work arrangements become more common (Caligiuri et al., 2020). Given the growing need for psychological safety, it is possible that some will no longer continue as entrepreneurs in the future, especially if remote work enables a similar lifestyle. Consequently, they may become more anchored to a location and to an organisation. This raises a fundamental question: Do they remain cosmopolitans, or do they start to develop a novel type of identity? Is it possible that the core values of an individual can change permanently after an external shock? In our opinion, this certainly deserves further investigation.

Overall, we have seen a change from open to closed, both in the world and in reflections of it in cosmopolitan entrepreneurs. However, in terms of research, we have only scratched the surface. Whether the behavioural change is permanent and experienced on a deeper attitudinal level while affecting the social identity of the cosmopolitan entrepreneur is not yet known. It is well understood that the evolution of an identity is a slow process and often takes years. This will certainly be a topic worth investigating in the future.

## Notes

1. In line with Paasi (1996), we understand ‘place’ as an abstraction referring to the cumulative archive of personal experiences and meanings which individuals gain from different locations during their lives.

2. We interviewed 25 Finnish-born cosmopolitans in spring 2020. When it came to our selection of interviewees, we sought variety in terms of gender, age and marital status. For more about the project, see https://www.utu.fi/en/university/turku-school-of-economics/international-business/research
